# Supplementary material for: Pharmacological targeting of MCL-1 promotes mitophagy and improves disease pathologies in an Alzheimer’s disease mouse model
Source: Nat Commun. 2020 Nov 12;11:5731. doi: 10.1038/s41467-020-19547-6 (PMC7665171; doi:10.1038/s41467-020-19547-6)
Supplement: Supplementary file 1 — Supplementary Information [file 41467_2020_19547_MOESM1_ESM.pdf]

Pharmacological targeting of MCL-1 promotes mitophagy and improves disease pathologies in an Alzheimer's disease mouse model

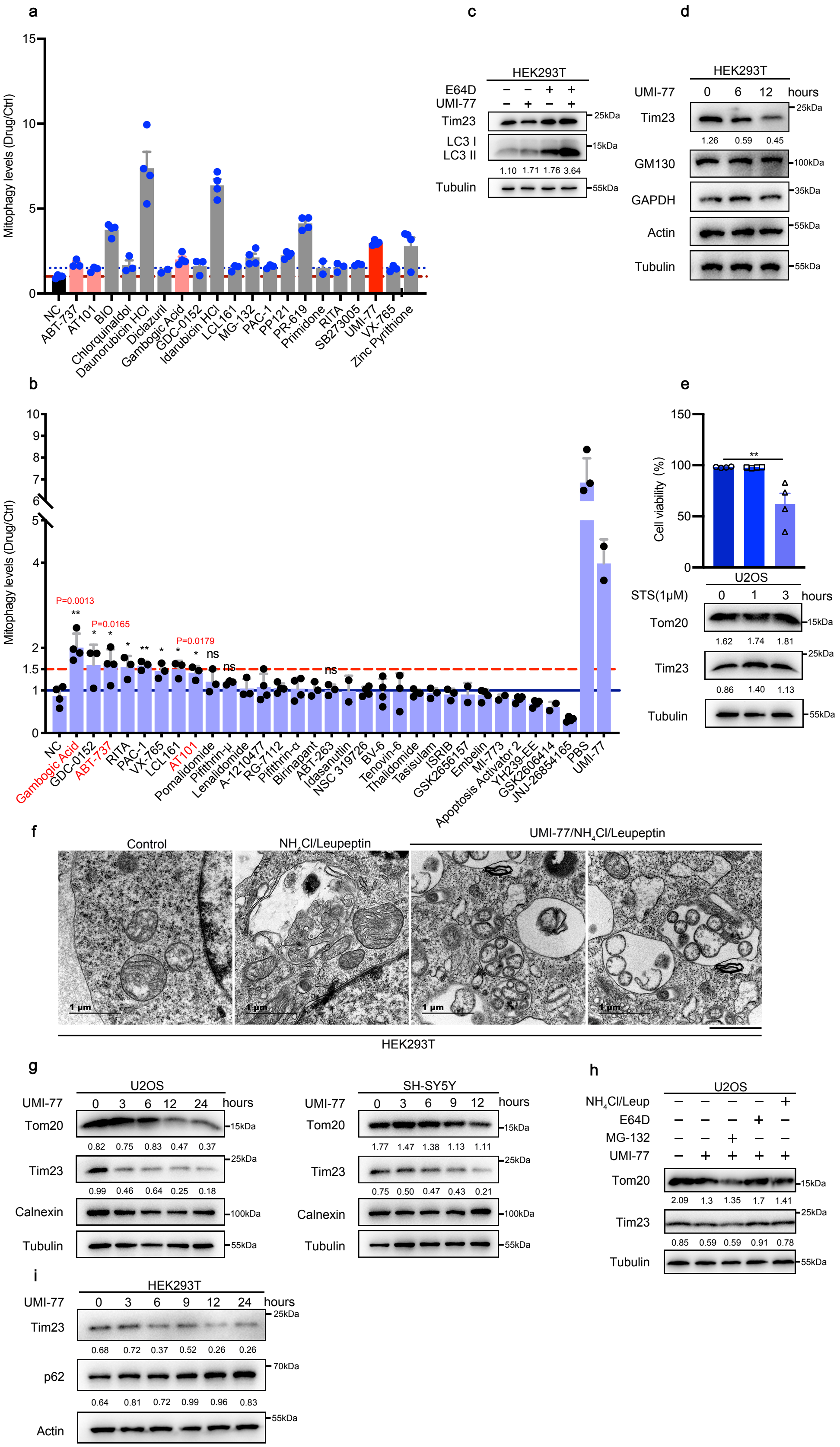

a
Mitophagy levels (Drug/Ctrl)
NC
ABT-737
AT101
BIO
Chloroquinaldol
Daunorubicin HCl
Diclazuril
Gambogic Acid
GDC-0152
Idarubicin HCl
LCL161
MG-132
PAC-1
PP121
PR-619
Primidone
RITA
SB273005
UMI-77
VX-765
Zinc Pyrithione
c
HEK293T
E64D
UMI-77
Tim23
LC3 I
LC3 II
Tubulin
1.10 1.71 1.76 3.64
25kDa
15kDa
55kDa
d
HEK293T
UMI-77 0 6 12 hours
Tim23
1.26 0.59 0.45
GM130
GAPDH
Actin
Tubulin
25kDa
100kDa
35kDa
55kDa
55kDa
b
Mitophagy levels (Drug/Ctrl)
P=0.0013
P=0.0165
P=0.0179
ns
NC
Gambogic Acid
GDC-0152
ABT-737
RITA
PAC-1
VX-765
LCL161
AT101
Pomalidomide
Pifithrin-μ
Lenalidomide
A-1210477
RG-7112
Pifithrin-α
Birinapant
ABT-263
Idasanutlin
NSC 319726
BV-6
Tenovin-6
Thalidomide
Tasisulam
ISRIB
GSK2656157
Embelin
MI-773
Apoptosis Activator 2
YH239-EE
GSK2606414
JNJ-26854165
PBS
UMI-77
e
Cell viability (%)
U2OS
STS(1μM) 0 1 3 hours
Tom20
1.62 1.74 1.81
Tim23
0.86 1.40 1.13
Tubulin
15kDa
25kDa
55kDa
f
Control
NH4Cl/Leupeptin
UMI-77/NH4Cl/Leupeptin
1 μm
HEK293T
g
U2OS
UMI-77 0 3 6 12 24 hours
Tom20
0.82 0.75 0.83 0.47 0.37
Tim23
0.99 0.46 0.64 0.25 0.18
Calnexin
Tubulin
15kDa
25kDa
100kDa
55kDa
SH-SY5Y
UMI-77 0 3 6 9 12 hours
Tom20
1.77 1.47 1.38 1.13 1.11
Tim23
0.75 0.50 0.47 0.43 0.21
Calnexin
Tubulin
h
U2OS
NH4Cl/Leup
E64D
MG-132
UMI-77
Tom20
2.09 1.3 1.35 1.7 1.41
Tim23
0.85 0.59 0.59 0.91 0.78
Tubulin
i
HEK293T
UMI-77 0 3 6 9 12 24 hours
Tim23
0.68 0.72 0.37 0.52 0.26 0.26
p62
0.64 0.81 0.72 0.99 0.96 0.83
Actin
25kDa
70kDa
55kDa

Supplementary Fig. 1. UMI-77 induces mitochondrial degradation through the lysosomal pathway.

a Twenty positive compounds were re-validated in HEK293T-mt-Keima cells. Bcl-2 family protein inhibitors were indicated in red. **b** Pro-apoptotic compounds were re-validated in HEK293T mt-Keima cells, with three replicates per drug. The mitophagy levels were analyzed using two-tailed t-test (data represent mean ± S.E.M.; n=3. *** $p<0.001$, ** $p<0.01$, * $p<0.05$). PBS was used as a positive control to induce autophagy. **c** HEK293T cells were treated with UMI-77 and E64D, cell lysates were immunoblotted with indicated antibodies. The numbers under the blots represent the gray scale quantification (LC3II/Tubulin). **d** HEK293T cells were treated with 5 μM UMI-77 for the indicated times and cell lysates were immunoblotted with indicated antibodies. The numbers under the blots represent the gray scale quantification (Tim23/Tubulin). **e** U2OS cells were treated with 1 μM staurosporine (STS) for the indicated times, cell lysates were immunoblotted with indicated antibodies and cell viability were estimated by using LIVE/DEAD™ cell imaging kit . Data were analyzed by one-way ANOVA (data represent mean ± S.E.M.; n=4. ** $p<0.01$ ($P=0.0062$)). The numbers under the blots represent the gray scale quantification (Tom20/Tubulin, Tim23/Tubulin). **f** HEK293T cells were treated with UMI-77 (5 μM) for 24 h, treated with NH_4Cl (20 mM) and Leupeptin (100nM) for 12 h, and the analysis by electron microscopy was performed. Scale bars, 1 μm. **g** U2OS and SH-SY5Y cells were treated with 5 μM UMI-77 for the indicated times and cell lysates were immunoblotted with indicated antibodies. The numbers under the blots represent the gray scale quantification (Tom20/Tubulin, Tim23/Tubulin). **h** U2OS cells were treated with 5 μM UMI-77 in the presence or absence of MG-132, E64D and NH_4Cl/Leupeptin (Leup) for 12 h, and cell lysates were immunoblotted with indicated antibodies. The numbers under the blots represent the gray scale quantification (Tom20/Tubulin, Tim23/Tubulin). **i** HEK293T cells were treated with 5 μM UMI-77 for the indicated times, and cell lysates were immunoblotted with indicated antibodies. The numbers under the blots represent the gray scale quantification (Tim23/Tubulin, p62/Tubulin). Source data are provided as a Source Data file.

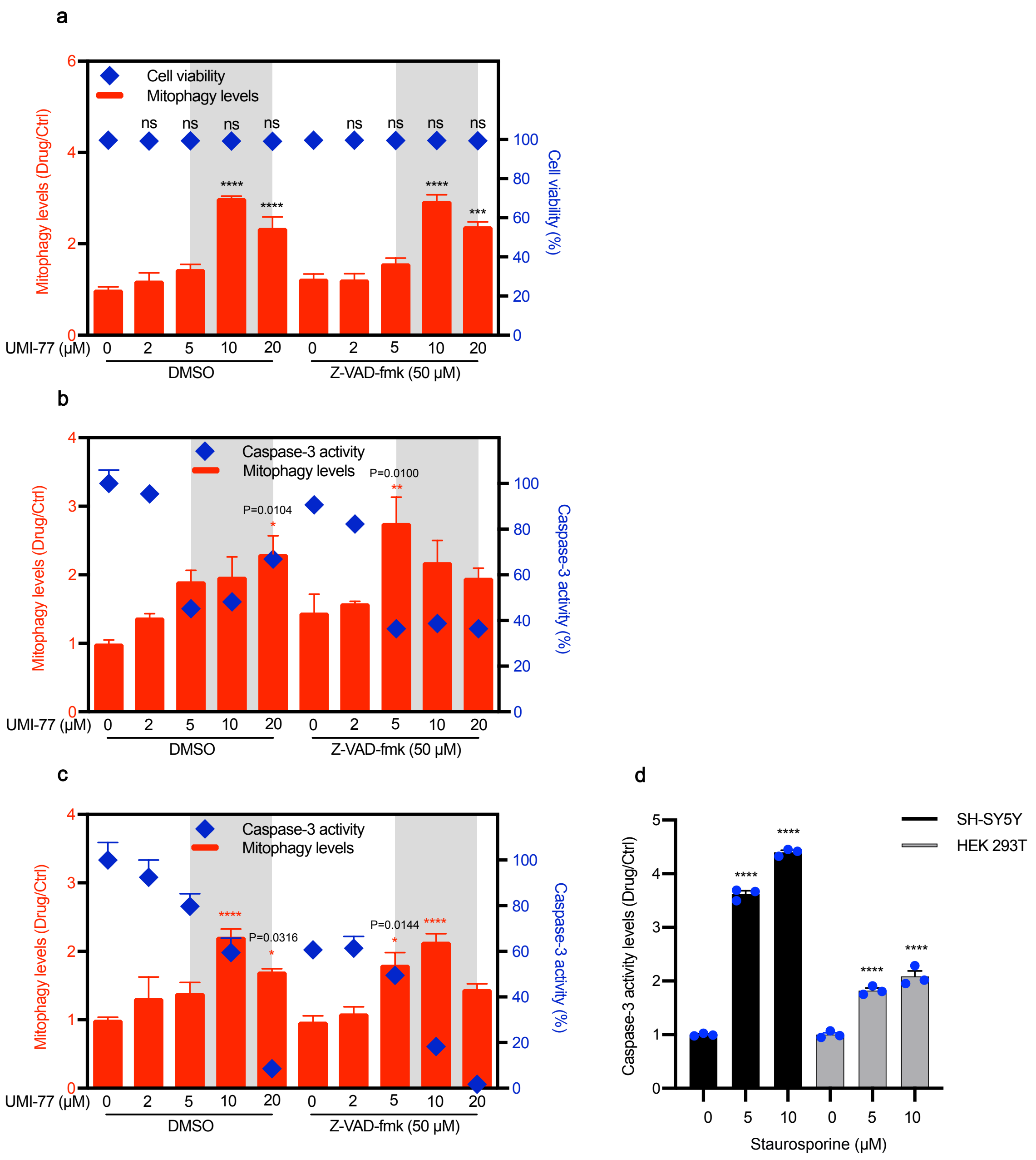

a
Cell viability
Mitophagy levels
Mitophagy levels (Drug/Ctrl)
Cell viability (%)
UMI-77 (μM)
DMSO
Z-VAD-fmk (50 μM)
b
Caspase-3 activity
Mitophagy levels
P=0.0104
P=0.0100
Caspase-3 activity (%)
c
P=0.0316
P=0.0144
d
SH-SY5Y
HEK 293T
Caspase-3 activity levels (Drug/Ctrl)
Staurosporine (μM)

Supplementary Fig. 2. UMI-77 induces mitophagy independent of apoptosis.

a SH-SY5Y cells were transfected with pcDNA3.1-mt-Keima plasmid for 24 h and treated with UMI-77 (0 μM, 2 μM, 5 μM, 10 μM, 20 μM) with or without Z-VAD-fmk (50 μM) for 12 h. Cell viability was estimated by using LIVE/DEAD™ cell imaging kit. One-way ANOVA (mean ± S.E.M.; n=4. **** $p<0.0001$, *** $p<0.001$. ns, not significant). **b** HEK293T cells were transfected with pcDNA3.1-mt-Keima plasmid for 24 h and treated with UMI-77 (0 μM, 2 μM, 5 μM, 10 μM, 20 μM) with or without Z-VAD-fmk (50 μM) for 24 h. Apoptosis levels was estimated using Caspase-Glo® 3/7 Assay. One-way ANOVA (data represent mean ± S.E.M.; n=4. ** $p<0.01$, * $p<0.05$). **c** As in (**b**), except SH-SY5Y cells were used. Apoptosis levels was estimated using Caspase-Glo® 3/7 Assay. One-way ANOVA (data represent mean ± S.E.M.; n=4. **** $p<0.0001$, * $p<0.05$). **d** Cells were treated with staurosporine (0 μM, 5 μM, 10 μM) for 3 h. Apoptosis levels were estimated as in (**b**). One-way ANOVA (data represent mean ± S.E.M.; n=4. **** $p<0.0001$). Source data are provided as a Source Data file.

a

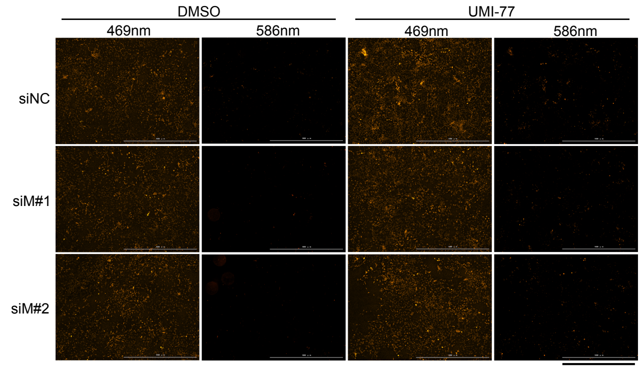
DMSO
UMI-77
469nm
586nm
469nm
586nm
siNC
siM#1
siM#2

b

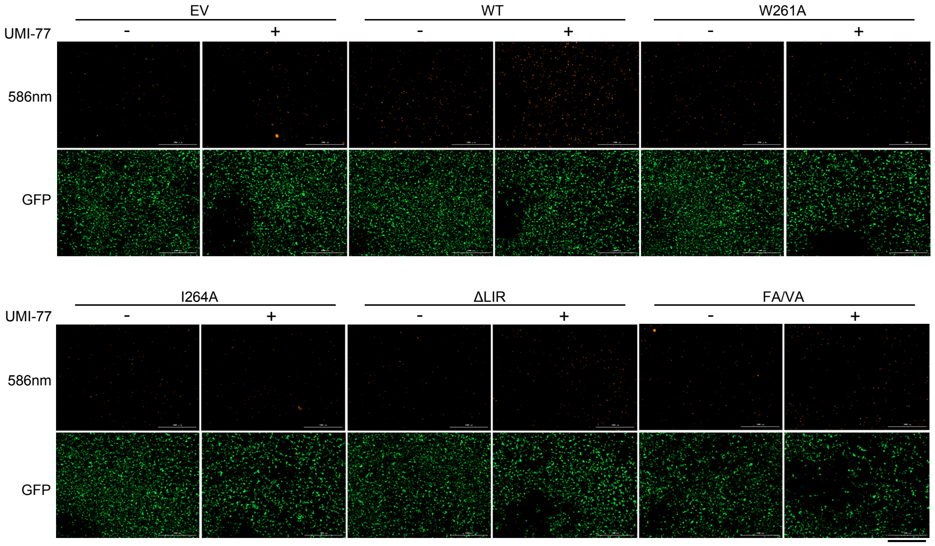
EV
WT
W261A
UMI-77
-
+
-
+
-
+
586nm
GFP
I264A
ΔLIR
FA/VA
UMI-77
-
+
-
+
-
+
586nm
GFP

c

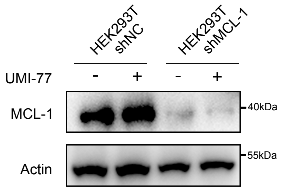
HEK293T shNC
HEK293T shMCL-1
UMI-77
-
+
-
+
MCL-1
40kDa
Actin
55kDa

d

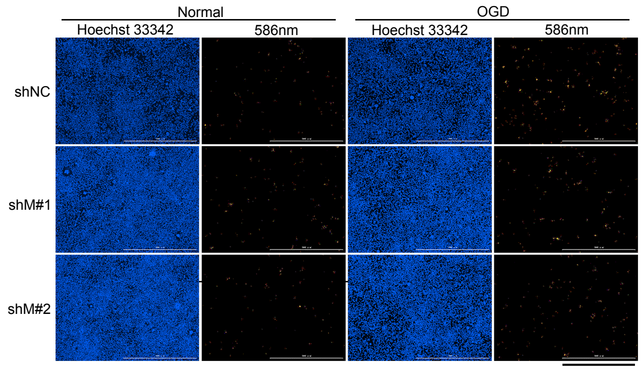
Normal
OGD
Hoechst 33342
586nm
Hoechst 33342
586nm
shNC
shM#1
shM#2

Supplementary Fig. 3. Representative images of Figure 2b, 3g, 5a.

a Representative image of Figure 2b. 469nm shows mitochondria and 586nm shows mitochondria in lysosome. **b** Representative image of Figure 3g. GFP shows MCL-1 knockdown cells and 586nm shows mitochondria in lysosome.

c HEK293T-MCL-1-knockdown (HEK293T-shMCL-1) and HEK293T-control-knockdown cells (HEK293T-shNC) were treated with UMI-77 as Figure 3g shown and cell lysates were immunoblotted with indicated antibodies. Source data are provided as a Source Data file. **d** Representative image of Figure 5a. Hoechst 33342 shows nucleus and 586nm shows mitochondria in lysosome. OGD: oxygen-glucose deprivation.

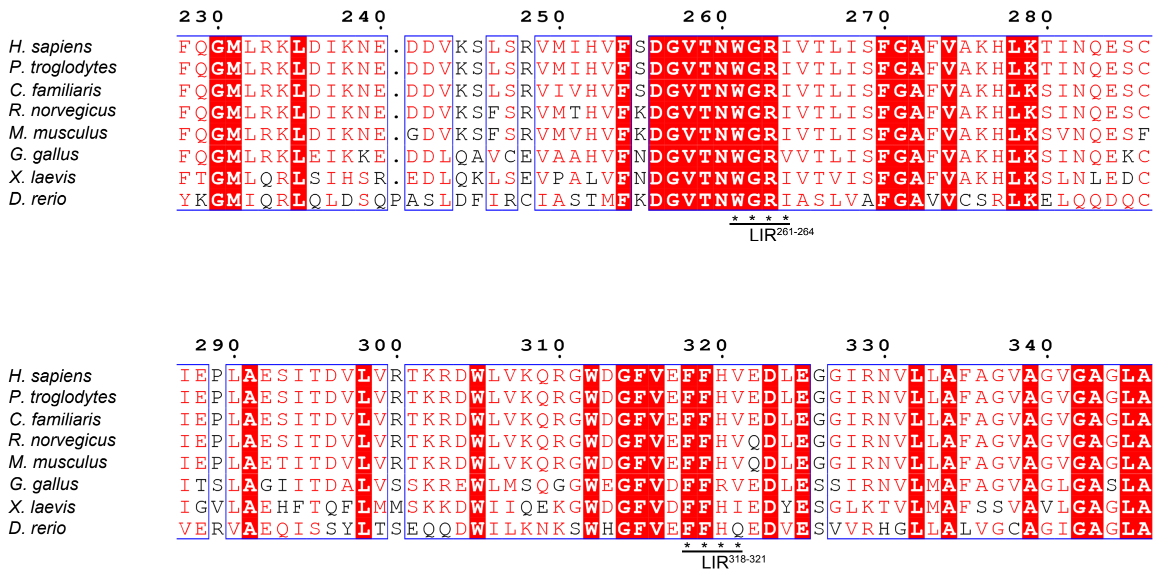

Supplementary Fig. 4. The LIR$^{261\text{-}264}$ motif of MCL-1 is strongly conserved. MCL-1 amino acid sequences were obtained from NCBI and aligned using clustalW software (http://www.genome.jp/tools-bin/clustalw). The image was generated using ESpripr 3.0 (http://espript.ibcp.fr/ESPript/cgi-bin/ESPript.cgi).

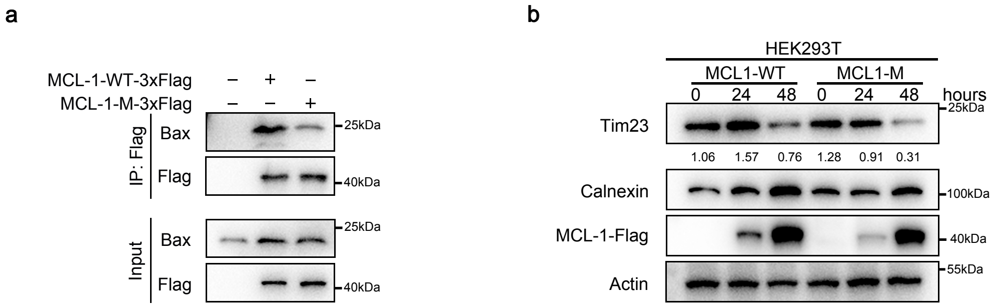

Supplementary Fig. 5. The L213A/D218A mutant of MCL-1 promotes mitophagy.
a HEK293T cells were transfected with MCL-1-WT-3xFlag or MCL-1-M-3xFlag (L213A/D218A) for 24 h, and the interaction between MCL-1 and Bax was analyzed by immunoprecipitation. **b** HEK293T cells were transfected with pcDNA3.1-MCL-1-WT (wild-type) or pcDNA3.1-MCL-1-M (L213A/D218A) plasmid for the indicated times and cell lysates were immunoblotted with indicated antibodies. The numbers under the blots represent the gray scale quantification (Tim23/Tubulin). Source data are provided as a Source Data file.

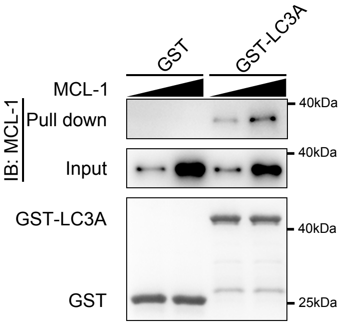

Supplementary Fig. 6. MCL-1 directly interacts with LC3A. Top two panels: Indicated purified proteins were incubated *in vitro* for two hours and GST pull-down was performed and analyzed by western blotting. Bottom panel: Coomassie staining was used to visualize GST-LC3A and GST proteins in the pull-down samples. Source data are provided as a Source Data file.

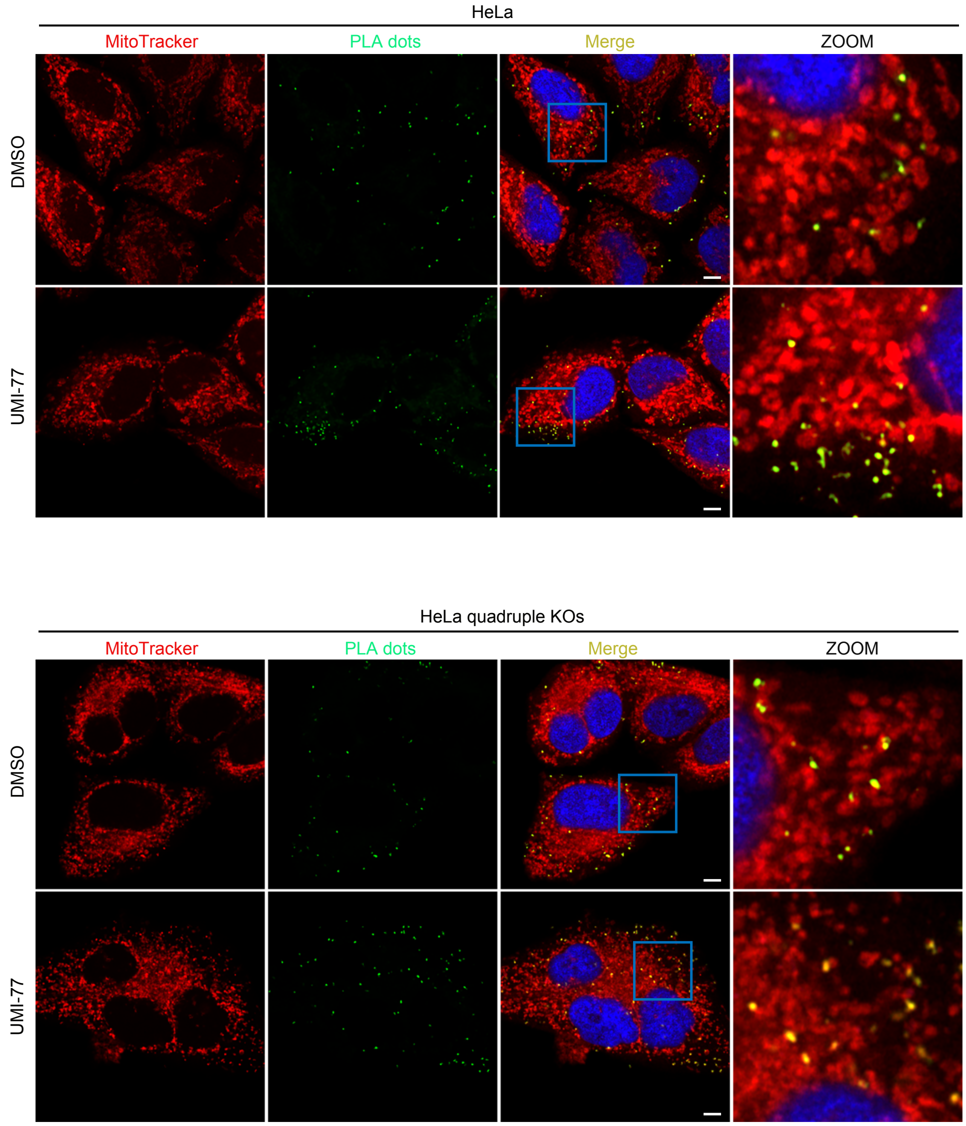

Supplementary Fig. 7. MCL-1 interacts with LC3A on mitochondria. Wild-type and quadruple KOs (NBR1, TAX1BP1, p62, and NDP52 knockout) HeLa cells were treated with UMI-77 (5 μM) for 3h and a PLA assay for MCL-1 and LC3A was performed. Cells were counter-stained with MitoTracker™ Deep Red FM. Scale bar, 5μm. Source data are provided as a Source Data file.

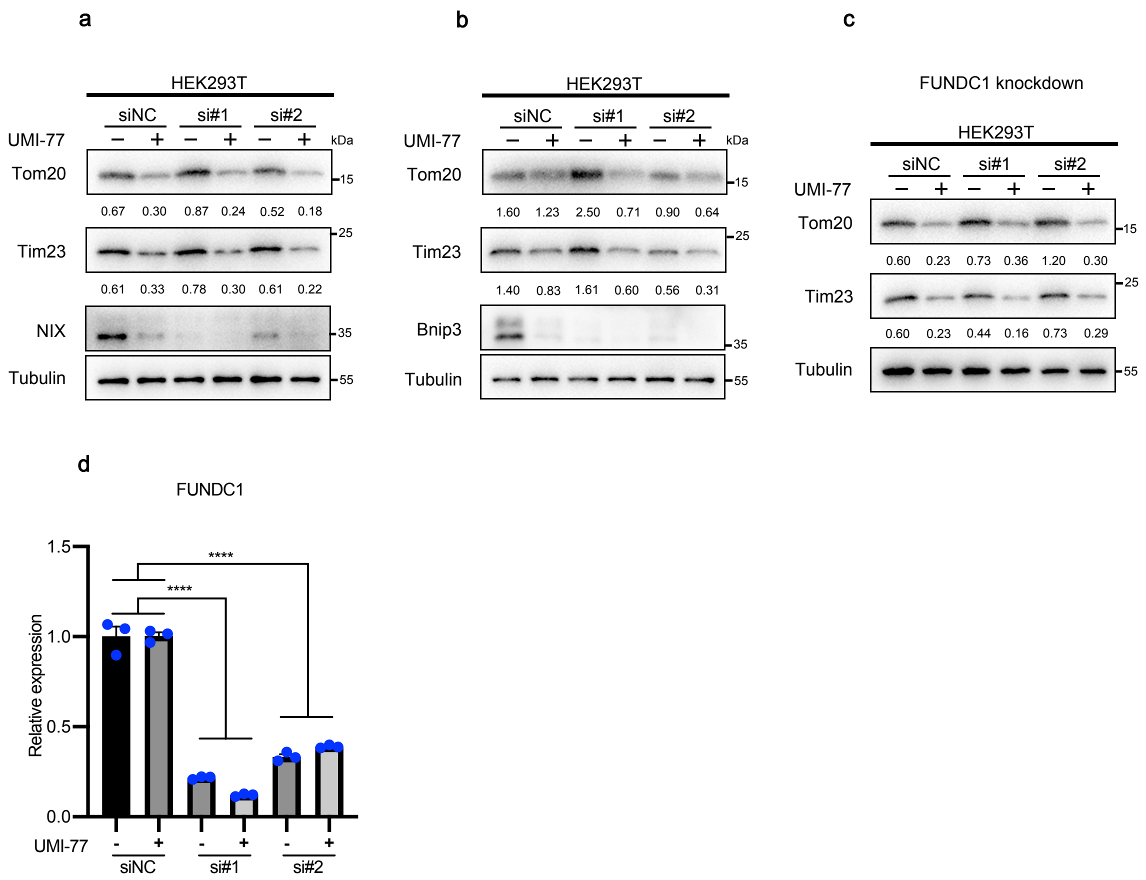

Supplementary Fig. 8. UMI-77 induces mitophagy independent of BNIP3, NIX, FUNDC1.

a HEK293T cells were transfected with NIX siRNA for 48 h and treated with 5 μM UMI-77 for 12 h. Cell lysates were immunoblotted for mitochondrial marker proteins (Tom20, Tim23). The numbers under the blots represent the gray scale quantification (Tom20/Tubulin, Tim23/Tubulin). siNC: scrambled siRNA.

b As in (**a**), except Bnip3 siRNA was used.

c As in (**a**), except FUNDC1 siRNA was used.

d Cells were treated as in (**c**) and total RNA were extracted by FastPure® Cell/Tissue Total RNA Isolation Kit for FUNDC1 qPCR. Data were analyzed using one-way ANOVA (data represent mean ± S.E.M.; n=3. **** $p<0.0001$). siNC: scrambled siRNA. Source data are provided as a Source Data file.

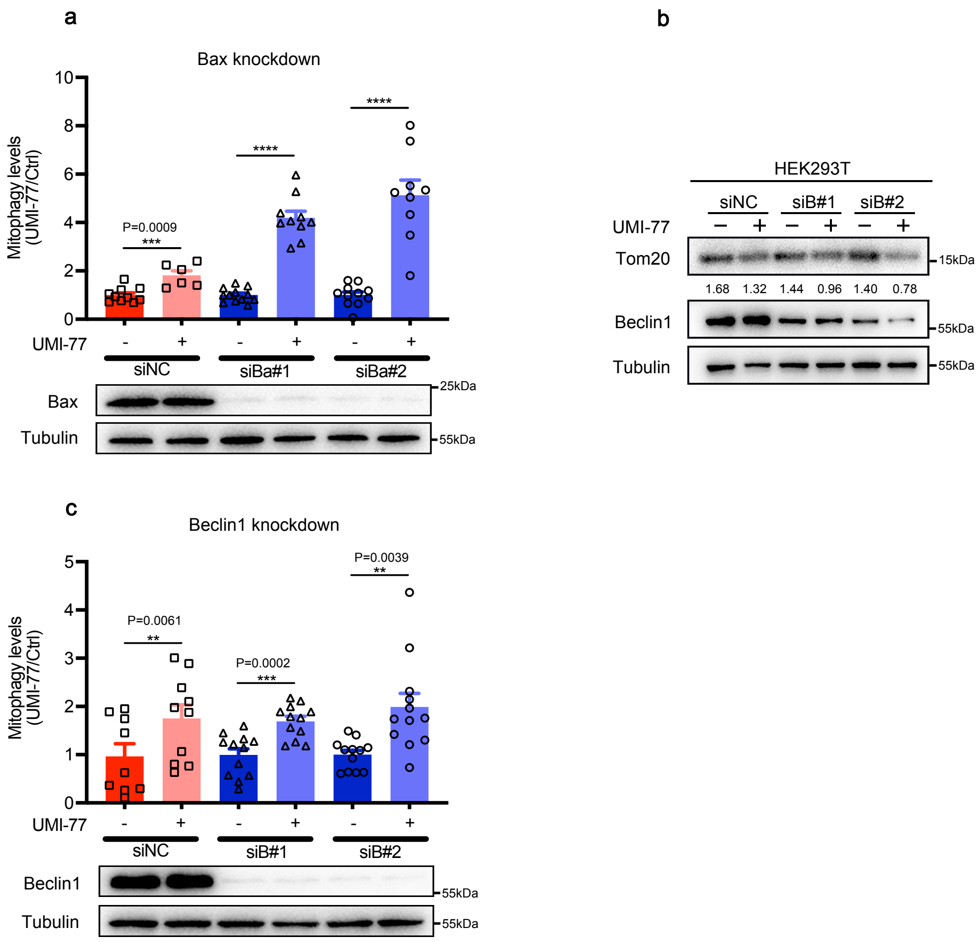

Supplementary Fig. 9. UMI-77 induces mitophagy independent of Bax and Beclin1.

a Bax knockdown HEK293T-mt-Keima cells were treated with 5 μM UMI-77 for 12 h. The mitophagy levels were analyzed using two-tailed t-test (data represent mean ± S.E.M.; The sample size was, in turn, n=10, n=6, n=12, n=12, n=10, n=11, n=9. *** p<0.001, **** p<0.0001.). The siRNA knockdown efficiency was shown using western blot. siNC: scrambled siRNA. **b** HEK293T cells were transfected with Beclin1 siRNA for 48 h and treated with 5 μM UMI-77 for 12 h. Cell lysates were immunoblotted for mitochondrial marker proteins (Tom20). The numbers under the blots represent the gray scale quantification (Tom20/Tubulin). siNC: scrambled siRNA. **c** Beclin1 knockdown HEK293T-mt-Keima cells were treated with 5 μM UMI-77 for 12 h. The mitophagy levels were analyzed using two-tailed t-test (data represent mean ± S.E.M.; n=11. ** p<0.01, *** p<0.001.). The siRNA knockdown efficiency was shown using western blotting. siNC: scrambled siRNA. Source data are provided as a Source Data file.

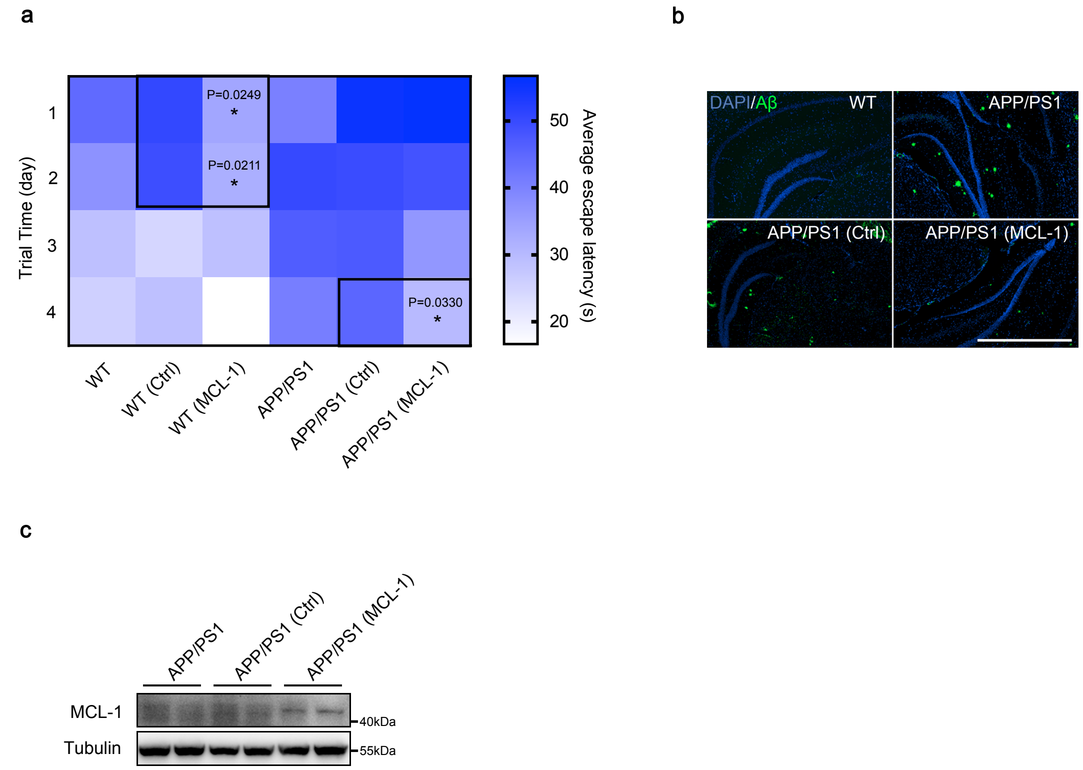

Supplementary Fig. 10. Overexpression of MCL-1 ameliorates cognitive decline in the APP/PS1 mouse model of Alzheimer's disease.

a Six-month-old C57BL/6 and APP/PS1 mice were overexpressed MCL-1 in hippocampus for one month. Latency to escape to a hidden platform in the Morris water maze during a 4-day training period ((WT, n=6), (WT (Ctrl vector), n=5), (WT (MCL-1 overexpression), n=6), (APP/PS1, n=6), (APP/PS1 (Ctrl vector), n=6), (APP/PS1 (MCL-1 overexpression), n=5). * $p<0.05$, two-tailed t-test). **b** Mice were treated as in (**a**) and immunohistochemistry of hippocampus was performed to stain for amyloid plaques (6E10 antibody, green) and nuclei (DAPI, blue). Scale bar, 1000μm. **c** Mice were treated as in (**a**) and brain lysates were immunoblotted for MCL-1 and Tubulin. Source data are provided as a Source Data file.

Supplementary Table 1.

Primers used in this study.

Primers for pCDH-mt-Keima	
pCDH-mt-Keima-F	5'-CCGGAATTCGAAATGCTGAGCCTGCGCCAGAG-3'
pCDH-mt-Keima-R	5'-CGCGGATCCTCAACCGAGCAAAGAGTGGC-3'
Primers for MCL-1 mutant	
W261A-F	5'-TCAGCGACGGCGTAACAAACGCGGGCAGGATTGTGACTCTCAT-3'
W261A-R	5'-ATGAGAGTCACAATCCTGCCCGCGTTTGTTACGCCGTCGCTGA-3'
I264A-F	5'-GCGTAACAAACTGGGGCAGGGCTGTGACTCTCATTTCTTTTGG-3'
I264A-R	5'-CCAAAAGAAATGAGAGTCACAGCCCTGCCCCAGTTTGTTACGC-3'
W261A/I264A-F	5'-TCAGCGACGGCGTAACAAACGCGGGCAGGGCTGTGACTCTCATTTCTTTTGG-3'
W261A/I264A-R	5'-CCAAAAGAAATGAGAGTCACAGCCCTGCCCGCGTTTGTTACGCCGTCGCTGA-3'
ΔLIR-F	5'-TCAGCGACGGCGTAACAAACGTGACTCTCATTTCTTTTGG-3'
ΔLIR-R	5'-CCAAAAGAAATGAGAGTCACGTTTGTTACGCCGTCGCTGA-3'
F318A/V321A-F	5'-GCTGGGATGGGTTTGTGGAGGCCTTCCATGCAGAGGACCTAGAAGGTGGCAT-3'
F318A/V321A-R	5'-ATGCCACCTTCTAGGTCCTCTGCATGGAAGGCCTCCACAAACCCATCCCAGC-3'
Primers for GST pull down	
pET28a-mcl1-F	5'-TAGAAGCTTGCGGCCGCACT-3'
pET28a-mcl1-R	5'-TTCTAGGTCCTCTACATGGA-3'
GST-LC3A-F	5'-CGCGGATCCGATGCCCTCAGACCGGCCTTT-3'
GST-LC3A-R	5'-CCGCTCGAGTCAGAAGCCGAAGGTTTCCT-3'
